# The interactome of 2-Cys peroxiredoxins in *Plasmodium falciparum*

**DOI:** 10.1038/s41598-019-49841-3

**Published:** 2019-09-19

**Authors:** Christina Brandstaedter, Claire Delahunty, Susanne Schipper, Stefan Rahlfs, John R. Yates, Katja Becker

**Affiliations:** 1 Biochemistry and Molecular Biology, Interdisciplinary Research Centre, Justus Liebig University Giessen, Heinrich Buff Ring 26-32, 35392 Giessen, Germany; 2 Molecular Medicine, The Scripps Research Institute, 10550 North Torrey Pines Rd., SR11, La Jolla, CA 92037 USA

**Keywords:** Oxidoreductases, Parasite physiology

## Abstract

Peroxiredoxins (Prxs) are crucially involved in maintaining intracellular H_2_O_2_ homeostasis via their peroxidase activity. However, more recently, this class of proteins was found to also transmit oxidizing equivalents to selected downstream proteins, which suggests an important function of Prxs in the regulation of cellular protein redox relays. Using a pull-down assay based on mixed disulfide fishing, we characterized the thiol-dependent interactome of cytosolic Prx1a and mitochondrial Prx1m from the apicomplexan malaria parasite *Plasmodium falciparum* (*Pf*). Here, 127 cytosolic and 20 mitochondrial proteins that are components of essential cellular processes were found to interact with *Pf*Prx1a and *Pf*Prx1m, respectively. Notably, our data obtained with active-site mutants suggests that reducing equivalents might also be transferred from Prxs to target proteins. Initial functional analyses indicated that the interaction with Prx can strongly impact the activity of target proteins. The results provide initial insights into the interactome of Prxs at the level of a eukaryotic whole cell proteome. Furthermore, they contribute to our understanding of redox regulatory principles and thiol-dependent redox relays of Prxs in subcellular compartments.

## Introduction

Research on peroxiredoxins (Prxs) has expanded rapidly in recent years^1^. After initial discovery in human erythrocytes^2,3^, Prxs—as cysteine-dependent peroxidases and sensor transducers—were described in all kingdoms of life including protozoal parasites such as *Toxoplasma*, *Trypanosoma*, *Leishmania*, and *Plasmodium*^4,5^. Prxs (EC 1.11.1.15) are ubiquitous cysteine-dependent peroxidases and can regulate the intracellular messenger function of H_2_O_2_. They are able to reduce endogenous and exogenous H_2_O_2_, peroxynitrite (ONOO^−^), and organic hydroperoxides (ROOH)^6^, and their recycling depends on redoxins such as thioredoxin (Trx) or glutaredoxin (Grx). In spite of the presence of catalase and glutathione peroxidase in most eukaryotic cells, the high abundance of Prxs (1% or more of cellular proteins) defines their role as the predominant cellular peroxide-reducing enzymes^7^. Prxs can be found in the cytosol, mitochondria, nucleus, and even plastids. They can interact with and inhibit the function of certain oncoproteins^8,9^, can enhance the toxic effect of natural killer cells^10^, maintain genome stability, promote longevity^11^, and can be induced by proliferative stimuli^12^, nitric oxide^13^, and oxidative stress^14^.

Peroxiredoxins are subdivided into six evolutionary clusters or subfamilies (Prx1, Prx5, Prx6, Tpx, PrxQ, and AhpE)^15,16^. Furthermore, Prxs can be categorized mechanistically into three subfamilies and—based on the number of active site cysteines involved in the catalytic cycle—into 1-Cys and 2-Cys peroxiredoxins. The 2-Cys Prxs can be further subdivided into typical and atypical 2-Cys Prxs. 1-Cys Prxs contain only the highly conserved peroxidatic cysteine (C_P_) in their N-terminal region but no resolving cysteine (C_R_) and can be reduced by small molecules such as glutathione (GSH). 2-Cys Prxs contain, along with the C_P_, the C_R_. Typical 2-Cys Prxs form a stable disulfide bond between the oxidized C_P_ and the C_R_ of the second subunit (intermolecular), and the atypical 2-Cys Prxs build a disulfide with the C_P_ in the same polypeptide chain (intramolecular) in the resolving step^17^.

During its complex life cycle, the unicellular eukaryotic parasite *Plasmodium falciparum* is continuously exposed to oxidative challenges resulting from its high metabolic rate, hemoglobin digestion, and the host immune system^18–22^. To maintain redox equilibrium, *P. falciparum* relies on a well-equipped antioxidant system including glutathione and thioredoxin-dependent proteins^23,24^, as well as superoxide dismutases. Notably, a genuine catalase and glutathione peroxidase are absent^25^. *P. falciparum* possesses five peroxiredoxins, which support parasite survival under enhanced oxidative stress and have been studied in detail, also as a model system to elucidate Prx functions and networks^26^. *Pf*Prx1a, located in the cytosol, belongs to the typical 2-Cys Prx family with its peroxidatic cysteine at position C50 and its resolving cysteine at position C170. It is constitutively expressed during the complete life cycle of *Pf* ^27^ and reduces not only H_2_O_2_^28^ but also ONOO^−^, *tert*-Butyl hydroperoxide (tBuOOH), and cumene hydroperoxide (CHP) by accepting *Pf* Trx and *Pf* plasmoredoxin (Plrx) as electron donors^29–31^. A *Pf*Prx1a knockout in *P. berghei* caused a defect in gametocyte formation^32^ and may be involved in development during multiplying stages such as sporozoite and exo-erythrocytic forms^33^. The mitochondrial *Pf* Prx1m is also a Trx-dependent member of the typical 2-Cys Prx family^34^. It contains its peroxidatic cysteine at position C67 and its resolving cysteine at position C187 and was crystallized by Boucher *et al*. (PDB 2C0D). Interestingly, *Plasmodium* is also able to import human Prx2^30^, further underscoring the importance of this protein family in the parasite host cell unit.

Prxs contribute to regulating intracellular H_2_O_2_ concentrations via their peroxidase activity. At persistently high H_2_O_2_ levels, the C_P_ can hyperoxidize, resulting in the interruption of the H_2_O_2_ reducing activity and the potential oxidation of other targets (floodgate hypothesis)^11,31^. Concerning this oxidation of target proteins, several concepts are presently discussed. The first hypothesis implies that Prx-controlled H_2_O_2_ levels can directly influence targeted proteins (TPs). However, since the reactivity of TPs towards H_2_O_2_ is five to seven orders of magnitude lower than the reactivity of a Prx towards H_2_O_2_, and the abundance of Prxs is much higher, TPs cannot successfully compete with a Prx for peroxides^5^. A second hypothesis for oxidation signaling is transduction of the signal from oxidized Prx to a selective downstream regulatory TP via thiol-disulfide exchange^5^. Such a mechanism has been proposed, for example, for the phosphatase PTEN^35^ for apoptosis signaling kinase 1 (ASK1)^36^, for MAPK phosphatases^35^, and the transcription factor STAT3, which can be activated by mammalian Prx2^37^. The third mechanism implies that oxidized Trx, which emerges from the recycling step in the catalytic cycle, can transfer the oxidation signal to a TP^36,38^.

To gain further insight in the thiol-disulfide exchange-based interactome of 2-Cys Prxs and obtain a more complete picture of Prx-dependent redox relays, we used N-terminally His-tagged *Pf* Prxs for a mixed disulfide fishing approach with whole cell extracts from malaria parasites and identified interacting proteins via mass spectrometry (MS).

## Results

**Figure 1:**
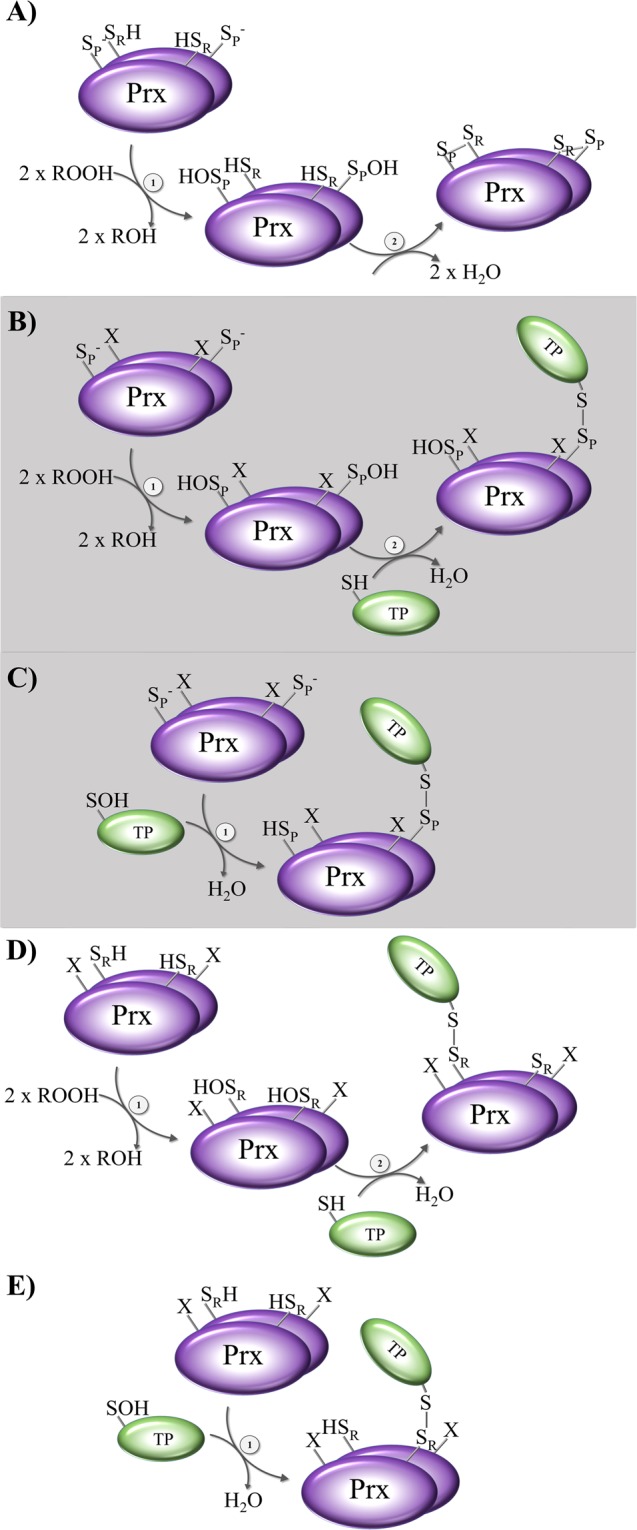
Proposed mechanisms of reduction and oxidation of targeted proteins via the experimentally used 2-Cys Prxs mutants. (**A**) The first two steps of peroxidation and resolution of the common catalytic cycle of typical 2-Cys Prx are shown: (1) peroxidation and (2) resolution with disulfide formation. (**B,C**) represent the proposed mechanisms of transmitting oxidizing or reducing equivalents via Prx resolving Cys mutants. (**D,E**) represent the proposed mechanisms of transmitting oxidizing or reducing equivalents via Prx peroxidatic Cys mutants. For further details, please see text.

In order to explain our experimental setup it is important to keep in mind the catalytic cycle of 2-Cys Prx (Fig. 1A). The cycle begins with the thiolate (S^−^) of the peroxidatic cysteine of reduced Prx. The Prx is now in the fully folded (FF) conformation and starts a nucleophilic attack at the peroxyl bond of the hydroperoxide substrate (ROOH), whereby the thiolate is oxidized to a sulfenic acid (SOH), and an alcohol is released (peroxidation). Under oxidative challenge, this sulfenic acid can be further oxidized to sulfinic acid (SO_2_H) (hyperoxidation) or even sulfonic acid (SO_3_H). In many organisms, enzymes such as the ATP-dependent sulfiredoxin (Srx) reduce sulfinic acid, thus reactivating (or rescuing) the hyperoxidized Prx. After sulfenic acid is formed at the C_P_, the Prx has to be recycled. The Prx switches its conformation from the FF to the locally unfolded (LU) conformation (resolution), and the C_R_ is able to form a disulfide bond with the C_P_, which is intermolecular in the typical 2-Cys Prxs and intramolecular in the atypical 2-Cys Prxs. Proteins such as Trx or Grx, which contain an attacking cysteine and a resolving cysteine (CxxC-motif), mediate the reduction of the disulfide. First, the attacking thiol(ate) of the reductant forms a mixed disulfide with the Prx, which is then reduced by the resolving cysteine of the reductant. After this recycling step, the Prx is reduced and activated for another catalytic cycle^11^ (recycling).

In our pull-down assay, proteins from trophozoite stage *P. falciparum* cell lysate were captured that interacted with immobilized wild type and active site Cys (C_R_ and C_P_) mutants of the two 2-Cys Prxs. In order to allow for these interactions—in a setting that is as physiological as possible—we exposed the Prxs (freshly reduced on the beads) to the freshly prepared cell lysates in their physiological redox state. The target proteins that had bound via disulfide bridges to the Prxs were then specifically eluted with DTT (Supp. Fig. 1). Only proteins that were identified in two or three out of the three independent biological experiments were taken into account.

Using the Prxs as bait, we identified 288 putatively interacting proteins for *Pf* Prx1a (10% of unknown function) (Table S1) and 547 proteins for *Pf* Prx1m (7% of unknown function) (Table S2), which are discussed in more detail below. The putative mechanisms of interaction of the target proteins with the C_R_ and the C_P_ mutants are shown in Fig. 1B,C and in Fig. 1D,E, respectively, and are discussed below.

### Identification of proteins interacting with *Pf* Prx1a

**Figure 2:**
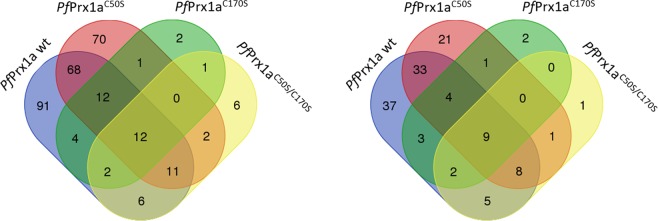
Venn diagrams of the number of proteins identified in the pull-down assay with *Pf* Prx1a. (**A**) Total number of proteins interacting via disulfide bridges with *Pf* Prx1a, *Pf* Prx1a^C170S^, *Pf* Prx1a^C50S^, or *Pf* Prx1a^C50S/C170S^. (**B**) Number of cytosolic proteins interacting via disulfides bridges with *Pf* Prx1a, *Pf* Prx1a^C170S^, *Pf* Prx1a^C50S^, or *Pf* Prx1a^C50S/C170S^.

When using the cytosolic *Pf* Prx1a as bait, we identified 91 proteins that exclusively interacted with *Pf* Prx1a wild type and 70 proteins that did so with *Pf* Prx1a^C50S^, in which the peroxidatic cysteine C50 had been mutated to a serine, and the resolving cysteine C170 was still present. Additionally, 68 proteins interacted with both wild type and *Pf* Prx1a^C50S^ (Fig. 2A), and two potentially interacting proteins were identified with the resolving cysteine mutant *Pf* Prx1a^C170S^. Proteins captured with the double mutant *Pf* Prx1a^C50S/C170S^ might reflect interacting partners caught exclusively with the remaining cysteine C74 of *Pf* Prx1a. C74 is too far away from the active site to be substantially involved in the catalytic mechanism of the protein. However, using other experimental approaches such as surface plasmon resonance, we already had observed a potential interaction between C74 and *Pf* Trx 1 (unpublished results). Additionally, TPs binding to the double mutant (and potentially also wt and single mutants) might originate from non-covalent interactions with the Prxs and elution when either oxidized Prx or oxidized TP becomes reduced and changes conformation, thus decreasing binding affinity. However, due to the extensive washing procedures included in our protocol, this possibility is unlikely to have a major impact on the results.

Figure 2B shows a Venn diagram in which only the numbers of *cytosolic* proteins interacting with the different *Pf* Prx1a variants are depicted (127 cytosolic TPs). This is important for further understanding functional redox relays in specific subcellular compartments (*Pf* Prx1a is a cytosolic protein). At the same time, the data suggests that the interaction of Prxs with TPs is not restricted to TPs from the same compartment but likely represents a phenomenon of relevance for several subcellular compartments. The overall distribution pattern in Fig. 2B is very similar to Fig. 2A, although the absolute numbers are lower. Again, most of the proteins were captured with *Pf* Prx1a wild type and the C_P_ mutant *Pf* Prx1a^C50S^ (37 and 21, respectively, with an overlap of 33 proteins). Therefore, the resolving C_R_ of *Pf* Prx1a is likely to be the Cys residue mainly driving protein–protein interactions (see also Fig. 1D,E), whereas the C_P_ is specialized in ROOH recognition. If the C_P_ is hyperoxidized, C_R_ could interact with TPs via a disulfide bond to transfer oxidizing or reducing equivalents or to protect the TP against hyperoxidation. Reductants such as thioredoxin could theoretically resolve this formed complex. To fully validate this hypothesis, further studies would be required.

Captured proteins are involved in various central cellular pathways such as carbohydrate or *S*-adenosylmethionine metabolism, protein folding, the translational machinery, or signal transduction. The TPs were clustered according to their function, summarized in Table 1, and are discussed in more detail below (see Table S1 and SI dataset *Pf*Prx1a for complete data).

**Table 1:** Functional clustering of cytosolic proteins interacting with *Pf*Prx1a.

*Functional cluster*	*Captured proteins*
**PfPrx1a wild type**	
Translation	40S ribosomal protein S23, 40S ribosomal protein S15A, 40S ribosomal protein S3A, eukaryotic initiation factor, translation initiation factor IF-2, nascent polypeptide-associated complex alpha chain, eukaryotic translation initiation factor 3 subunit 5, 40S ribosomal protein S18, 60S ribosomal protein L35ae, 40S ribosomal protein S21 (RPS21), eukaryotic translation initiation factor 3 subunit 8, eukaryotic translation initiation factor 3 subunit 10, glutamine-tRNA ligase, lysine-tRNA ligase (KRS1), 60S ribosomal protein L17, eukaryotic translation initiation factor 2 gamma subunit, 60S ribosomal protein L14
Protein degradation	26S proteasome regulatory subunit RPN11, 26S protease regulatory subunit 7, 26S protease regulatory subunit 10B, 26S protease regulatory subunit 8, 26S protease regulatory subunit 4, ubiquitin carboxyl-terminal hydrolase, proteasome regulatory protein
Protein folding	Heat shock protein 40, endoplasmin
Protein transport	Ras-related protein Rab-2 (RAB2), karyopherin alpha (KARalpha)
S-adenosylmethionine metabolism	S-adenosylmethionine decarboxylase/ornithine decarboxylase
Carbohydrate metabolism	Phosphoglycerate mutase
Signal transduction	Casein kinase 1 (CK1), protein phosphatase 2C (PP2C)
Others	Carbamoyl phosphate synthetase (cpsSII), acyl-CoA synthetase (ACS11), small GTP-binding protein sar1 (SAR1), serine repeat antigen 5 (SERA5)
**PfPrx1a** ^**C50S**^	
Translation	RNA pseudouridylate synthase, eukaryotic translation initiation factor 3 37.28 kDa subunit, serine-tRNA ligase, 60S ribosomal protein L11a, 40S ribosomal protein S10, 60S ribosomal protein L22, box C/D snoRNP rRNA 2’-O-methylation factor, U4/U6.U5 tri-snRNP-associated protein 2, alanine-tRNA ligase, glycine-tRNA ligase, histidine-tRNA ligase
Protein degradation	Proteasome subunit alpha type-4, ubiquitin domain-containing protein DSK2, 26S proteasome regulatory subunit RPN10, proteasome subunit alpha type-5, RING zinc finger protein
Protein folding	Peptidyl-prolyl cis-trans isomerase (CYP19B)
Protein transport	Protein transport protein SEC. 31, exportin-1, protein transport protein SEC. 13
**PfPrx1a** ^**C170S**^	
Translation	60S ribosomal protein L23
Carbohydrate metabolism	Deoxyribose-phosphate aldolase
**PfPrx1a** ^**C50S/C170S**^	
Translation	Elongation factor 1-beta (EF-1beta)
**PfPrx1a wild type, PfPrx1a** ^**C50S**^	
Translation	60S ribosomal protein P0 (PfP0), elongation factor 1-gamma, 60S ribosomal protein L6-2, 60S acidic ribosomal protein P2 (PfP2), 40S ribosomal protein S4, 60S acidic ribosomal protein P1, translation initiation factor 4E (eIF4E), asparagine-tRNA ligase, 40S ribosomal protein S5, 60S ribosomal protein L3 (RPL3), 40S ribosomal protein S3
Protein degradation	26S proteasome AAA-ATPase subunit RPT3, suppressor of kinetochore protein 1, ubiquitin carboxyl-terminal hydrolase 2
Protein folding	Heat shock protein 70 (HSP70-3), heat shock protein 70 (HSP70-2), protein disulfide isomerase (PDI-11)
Carbohydrate metabolism	Phosphoribosyl pyrophosphate synthetase, enolase (ENO), fructose-bisphosphate aldolase, L-lactate dehydrogenase (LDH), phosphoglycerate kinase
S-adenosylmethionine metabolism	S-adenosyl-L-homocysteine hydrolase (SAHH), phosphoethanolamine N-methyltransferase (PMT)
Others	Casein kinase 2, alpha subunit, 1-cys peroxiredoxins, dihydropteroate synthetase (DHPS), erythrocyte membrane-associated antigen, purine nucleoside phosphorylase (PNP), dolichyl-phosphate-mannose protein mannosyltransferase
**PfPrx1a wild type, PfPrx1a** ^**C170S**^	
Translation	40S ribosomal protein S7, 60S ribosomal protein L5
Purine metabolism	Inosine-5′-monophosphate dehydrogenase

Cytosolic proteins with known functions that were captured in the implemented pull-down assay were clustered depending on their metabolic role. Proteins captured with *Pf*Prx1a wt, the peroxidatic Cys mutant *Pf*Prx1a^C50S^, the resolving Cys mutant *Pf*Prx1a^C170S^, the double active site mutant *Pf*Prx1a^C50S/C170S^, and proteins that were captured with both *Pf*Prx1a wt and *Pf*Prx1a^C170S^ (overlap) are listed.

### Identification of proteins interacting with *Pf* Prx1m

**Figure 3:**
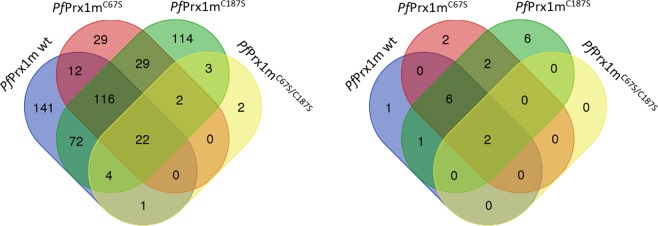
Venn diagrams of the number of proteins identified in the pull-down assay with *Pf*Prx1m. (**A**) Total number of proteins interacting via disulfide bridges with *Pf*Prx1m, *Pf*Prx1m^C187S^, *Pf*Prx1m^C67S^, or *Pf*Prx1m^C67S/C187S^. (**B**) Number of mitochondrial proteins interacting via disulfide bridges with *Pf*Prx1m, *Pf*Prx1m^C187S^, *Pf*Prx1m^C67S^, or *Pf*Prx1m^C67S/C187S^.

In mitochondria, oxidative post-translational modifications (Ox-PTMs) have been linked to mitochondrial dysfunctions and pathophysiological consequences such as heart failure, acute neuronal trauma, peripheral diseases, and chronic neurodegenerative diseases^39,40^. In malaria parasites, mitochondrial redox relays have hardly been studied so far. With our pull-down approach, we identified 547 proteins that interact with *Pf* Prx1m (Table S2). Figure 3A shows the number of proteins captured by *Pf* Prx1m wild type, the C_P_ mutant *Pf* Prx1m^C67S^, the C_R_ mutant *Pf* Prx1m^C187S^, and *Pf* Prx1m^C67S/C187S^, along with the overlap between these groups. Based on their presumed or known function, we identified among these target proteins 20 mitochondrial proteins, which are likely to represent the physiological interacting partners in *Plasmodium* (Fig. 3B). These are discussed in more detail below (see also Table 2, Table S2, and SI dataset *Pf* Prx1m for complete data). Proteins captured with the double active site mutant *Pf* Prx1m^C67S/C187S^ potentially interact via non-active site cysteines of *Pf* Prx1m (C54, C55, or C152), which seems to be irrelevant in the mitochondrion since no respective mitochondrial proteins were identified (Fig. 3B). Using *Pf* Prx1m wild type as bait, we identified one putatively interacting mitochondrial protein (Fig. 3B).

**Table 2:** Functional clustering of mitochondrial proteins interacting with *Pf*Prx1m.

*Functional cluster*	*Captured protein*
**PfPrx1m wild type**	
Lipid metabolism	Diacylglycerol kinase
**PfPrx1m** ^**C67S**^	
Protein folding	GrpE protein homolog, mitochondrial
Others	Sortilin
**PfPrx1m** ^**C187S**^	
Anti-oxidative stress system	Ferrodoxin reductase-like protein, superoxide dismutase [Fe] (FeSOD), glutathione S-transferase, glutathione reductase
Energy metabolism	ATP synthase subunit beta, mitochondrial
Others	Mitochondrial acidic protein MAM33
**PfPrx1m wild type, PfPrx1m** ^**C187S**^	
Others	Acyl-CoA synthetase (ACS10)

Mitochondrial proteins of known function that were captured in the implemented pull-down assay were clustered depending on their metabolic role. Proteins captured with *Pf*Prx1m wt, the peroxidatic Cys mutant *Pf*Prx1m^C67S^, the resolving Cys mutant *Pf*Prx1m^C187S^, the double active site mutant *Pf*Prx1m^C67S/C187S^, and proteins that were captured with both *Pf*Prx1m wt and *Pf*Prx1m^C187S^ (overlap) are listed.

### Validation of the pull-down assays and functional implications

**Figure 4:**
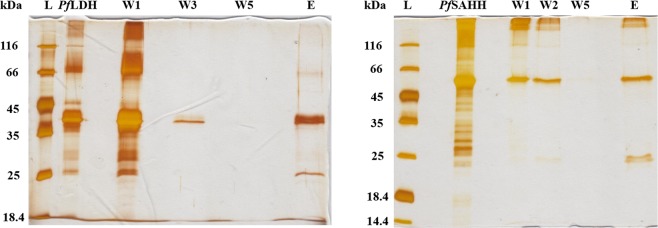
Validation of the interaction of immobilized *Pf*Prx1a wild type with recombinant *Pf*LDH and *Pf*SAHH. After incubation of *Pf*Prx1a with the proteins and extensive washing, *Pf*LDH and *Pf*SAHH could be specifically eluted with DTT. Eluates were separated on a 12% SDS-PAGE and silver stained. W: number of washing steps, E: eluate with 10 mM DTT.

In order to validate the results of the pull-down assays with an additional method, we recombinantly produced and purified two selected target proteins, namely plasmodial L-lactate dehydrogenase (*Pf* LDH) and *S*-adenosyl-L-homocysteine hydrolase (*Pf* SAHH). Both proteins had been detected as interaction partners of *Pf* Prx1a wild type and the C_P_ mutant *Pf* Prx1a^C50S^ in our pull-downs (Table 1 and SI Table 1). Recombinant *Pf* Prx1a wt was immobilized, reduced, and incubated with recombinant *Pf* LDH wt or *Pf* SAHH wt. After extensive washing, both proteins could indeed be specifically eluted by DTT, confirming them as disulfide-based binding partners of *Pf* Prx1a (see Fig. 4). In order to account for highly abundant sticky proteins, we compared our list to the mass spectrometry-based list of highly abundant proteins in *P. falciparum* trophozoites provided in the main tables of Pease *et al*.^41^. Based on this comparison, only lactate dehydrogenase and enolase might be unspecific binders. Since, however, a specific interaction between *Pf* Prx1a and these proteins cannot be ruled out and in fact an interaction was proven for LDH via gel electrophoresis (see Fig. 4), we decided to leave these two proteins in the catalog of potential target proteins.

**Figure 5:**
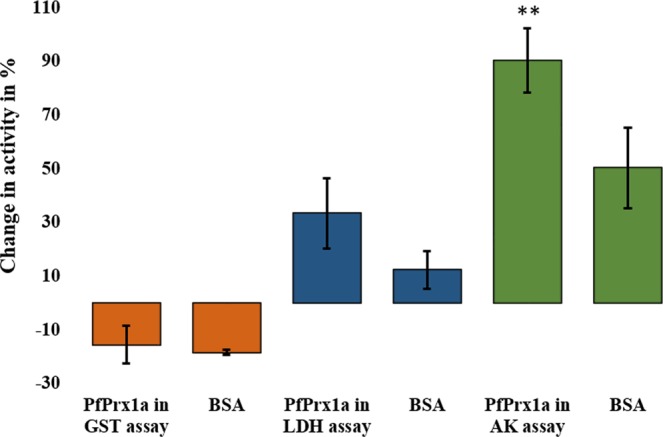
Influence of 25 µM reduced *Pf*Prx1a wt on the enzyme activities of *P. falciparum* glutathione *S*-transferase (GST), lactate dehydrogenase (LDH), and adenylate kinase (AK). The percentage of decrease or increase in activity of the Prx-free controls is shown. The influence of 25 µM BSA was tested as an additional control to account for unspecific stabilizing effects. A Student’s unpaired *t*-test with a 95% confidence level compared means of samples and controls. *p < 0.05, **p < 0.01, ***p < 0.001.

To gain initial insights into functional implications of the interaction between Prxs and their target proteins, we analyzed the influence of 25 µM reduced or oxidized *Pf* Prx1a wt on the enzyme activities of three selected recombinant (non-prereduced) *P. falciparum* enzymes for which we have robust assay systems established in our laboratory: glutathione-*S*-transferase (GST), lactate dehydrogenase (LDH), and adenylate kinase (AK). As additional controls, buffer alone was employed or 25 µM BSA to account for unspecific stabilizing effects. For GST both (reduced or oxidized) *Pf* Prx1a and BSA led to a slight, non-significant decrease in activity. In the LDH and AK assays, a clear activity increase was determined, which was higher for *Pf* Prx1a than for BSA and reached significance for the interaction between *Pf* Prx1a and adenylate kinase. Data is shown for the reduced Prx (Fig. 5). Interestingly, the oxidation state of the Prx did not have a major effect on the results. In the GST assay, we determined 8% activity loss when incubating with reduced Prx and 16% loss when incubating with oxidized Prx. LDH activity increased by 47% in the presence of reduced Prx and by 40% in the presence of oxidized Prx. AK activity increased by 78% when incubating with reduced Prx and by 72% after exposure to oxidized Prx. This data indicates that Prx can have a major effect on the activity of target enzymes identified by mixed disulfide fishing. However, in order to fully understand the flow of electrons and the respective mechanism of activation or inhibition further experiments are required in future.

## Discussion

Thiol-based redox relays play central roles in cellular reactions to oxidative challenge, as well as in redox regulatory and adaptive processes. In malaria, they are likely to be involved in propagation and stage conversion of the parasites, in the functionality of the parasite host cell unit, in the pathophysiology of the disease, and in mechanisms of drug action and resistance. Several pull-down approaches to study redox cascades in *P. falciparum* and other organisms have been made so far. They were based on active site cysteine mutants of Trx^42–48^, Grx^44,49^, and plasmoredoxin^44^ as bait proteins. Sturm *et al*., e.g., identified 33 putative interacting proteins from various metabolic pathways for Trx, Grx, and Plrx from whole cell *Plasmodium falciparum* parasite lysates^44^. In this and other studies, peroxiredoxins were found to interact with Trx via an intermolecular disulfide bond. However, the interactome of Prxs themselves has hardly been explored^4,5^. Recent studies suggest that Prxs can “transfer oxidizing equivalents” via a mixed disulfide to downstream signaling proteins, meaning that an oxidized protein A (in this case Prx) can receive electrons from a second protein B while transferring the oxidation signal (disulfide or sulfenic acid formation) from A to B. Selected examples include apoptosis signaling kinase 1, MAPK phosphatases, and the transcription factor STAT3 and are summarized in^5^). Such target proteins seem to react only slowly with hydrogen peroxide directly, although their activities depend on Cys oxidation^36,38,50^. Recent studies furthermore revealed that—to some extent—transient mixed disulfides between Prxs and targeted proteins are reducible by Trx, as monitored via a Trx-trapping mutant^45^.

### Mechanistic considerations concerning the experimental setup

The aim of our study was to gain insight into the complete interactome of 2-Cys-Prxs (which is not limited to Trx recognition) in distinct subcellular compartments (cytosol and mitochondrion) of malaria parasites. Employing the principle of mixed disulfide fishing^51^ with Prxs and their active site cysteine mutants, we aimed to identify proteins that interact with 2-Cys *Pf*Prxs via a transient or stable mixed disulfide bond. Although this *in vitro* approach represents a somewhat artificial system, we believe it can serve as an attractive model for studying cysteine-based interactions since stabilized disulfides are employed to capture interaction partners.

In our previous studies, using a glutathione biosensor comprising human glutaredoxin-1 linked to a redox-sensitive green fluorescent protein (hGrx1-roGFP2), the cytosolic basal glutathione-dependent redox potentials (E_GSH_) of 3D7 and Dd2 trophozoites were determined to be −314.2 mV and −313.9 mV, respectively, which is indicative of a highly reducing compartment^52^. This was confirmed in later studies with values of −309 mV in 3D7 parasites^53^ and −304 mV for the NF54 strain (after stable integration of the sensor)^54^. For the apicoplast and the mitochondrion of 3D7 trophozoites, E_GSH_ values of −267 mV and −328 mV, respectively, were obtained^53^. Therefore, under unstressed conditions, *P. falciparum* trophozoites show a highly reducing cytosol, in spite of intense hemoglobin digestion potentially leading to prooxidative by-products^20^. During the preparation of cell lysates in our experiment, the cytosol was most likely partially oxidized, allowing for reactions of (by then also partially oxidized) Prxs with downstream proteins under conditions of oxidative challenge. This implies that the Prxs can potentially act as both reducers and oxidizers of putatively interacting proteins. For identification of interaction partners, we used a qualitative shotgun proteomics approach, drawing conclusions about positively identified proteins that were present in at least two out of three independent experiments. While we did not make quantitative comparisons about identified peptides/proteins, spectral counts are included in the supplemental materials.

It should be kept in mind that, according to present knowledge, the midpoint reduction potentials measured for Prx proteins are quite high (measuring dithiol/disulfide forms)^55,56^, so even if they can interact with oxidized proteins, the equilibrium constant would typically favor reduced Prx and oxidized TP. Furthermore, the usual redox relay hypothesis implies that the TPs are largely unreactive with oxidants compared to Prxs, therefore favoring Prxs over TPs as reaction partners for H_2_O_2_. Nevertheless, based on the results of our interaction analyses, we would like to propose a concept describing Prxs as potential reductants. Possible reducing and oxidizing routes of 2-Cys Prxs and their mutants in our experimental setup are delineated in the following.

Figure 1A shows the steps of peroxidation and resolution of the common catalytic cycle of typical 2-Cys Prxs. Here, hydroperoxide substrates at the reactive peroxidatic Cys oxidize the fully reduced Prx (step 1), thereby forming a sulfenic acid at the C_P_. In the next step (step 2), the resolving Cys forms an intramolecular disulfide bond with the C_P_ by releasing H_2_O. Thioredoxin can then resolve this disulfide bond (not shown)^45^.

Resolving Cys mutants (see Fig. 1B,C) can theoretically act as oxidizers or reducers. C_R_ mutants are still highly reactive towards hydroperoxide substrates (Fig. 1B). Here, sulfenic acid formation is triggered in the same way as in wild type Prxs (step 1). However, an ensuing disulfide bond formation with the resolving Cys is not possible since the C_R_ has been mutated (to serine). The identification of proteins interacting with the remaining peroxidatic Cys supports the notion that in the next step (step 2) downstream regulatory signaling proteins can receive the oxidizing equivalents from the oxidized Prx by building a mixed disulfide intermediate. In case hydroperoxide substrates do not oxidize the 2-Cys Prx-resolving Cys mutant (Fig. 1C), the highly reactive reduced peroxidatic Cys might be able to interact with oxidized target proteins (TPs) (step 1) by building a mixed disulfide via a SOH or a disulfide on the TP. Afterwards, reduced redoxins such as Trx might resolve these disulfide bonds *in vivo*^45^, thereby releasing the reduced Prx and the TP (not shown).

The same might in principle be postulated for peroxidatic Cys mutants (Fig. 1D,E). When C_P_ mutants act as transducers of oxidizing signals, hydroperoxide substrates might oxidize the Prxs at the extant resolving Cys (step 1 in Fig. 1D). The sulfenic acid formed at the C_R_ (HOS_R_) might then be able to transmit the oxidizing signal to downstream interacting proteins by building a disulfide intermediate. Assuming that C_P_ mutants are acting as reducers, hydroperoxide substrates do not oxidize the remaining C_R_. Instead, the mutants reduce oxidized targeted proteins via mixed disulfide formation (Fig. 1E, step 1). As in the case of C_R_ mutants, downstream redoxins could resolve this disulfide bond *in vivo*. It should be noted here that the possibility of the oxidized protein (Fig. 1C,E), which Prx might reduce, possessing a disulfide bridge as a signal of oxidation rather than a sulfenic acid (not shown in the figure) cannot be excluded.

**Figure 6:**
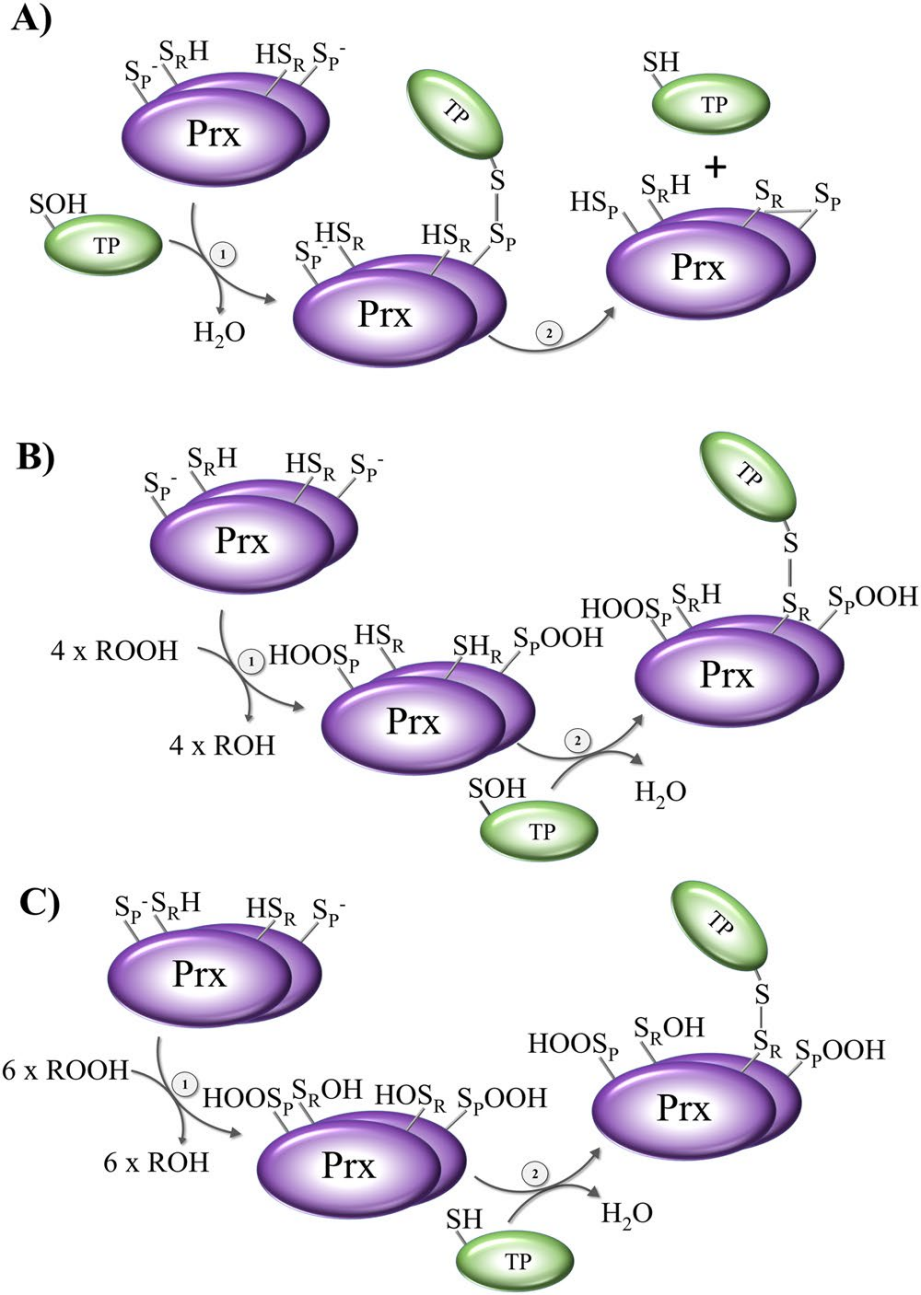
Proposed mechanisms of reduction and oxidation of targeted proteins via 2-Cys Prx wild type. (**A**) Reduction of oxidized targeted proteins (TP) via the Prx peroxidatic Cys: (1) reduction of the oxidized TP via the peroxidatic Cys of Prx and (2) resolving the mixed disulfide via the resolving Cys of Prx. (**B**) Reduction of oxidized TP via the Prx resolving Cys: (1) oxidation of the 2-Cys Prx and (2) reduction of oxidized TP via the resolving Cys of Prx. (**C**) Oxidation of reduced TP via the oxidized resolving Cys of Prx: (1) oxidation/hyperoxidation of Prx and (2) oxidation of the reduced TP via the resolving Cys of Prx.

For wt Prx we had assumed—based on the general principle of mixed disulfide fishing—that a TP binding to the C_P_ would be immediately released due to the presence of the resolving cysteine. However, in order to correct for proteins potentially binding to non-active site cysteines, we included the *Pf*Prx wt enzymes as controls. Interestingly, when evaluating our data, a rather large number of proteins that had exclusively bound to the Prx wt (and not, e.g., to the active site double mutant) were identified in all three independent experiments. Although an interaction with non-active site cysteines might still occur, we therefore would like to propose mechanisms that might allow for the transduction of reducing and oxidizing equivalents by wild type 2-Cys Prxs (*in vitro* and *in vivo*) and might also facilitate the formation of stable reaction intermediates. These mechanisms are depicted in Fig. 6 and discussed in the following.

### Implications for 2-Cys Prx wild type *in vivo*

Reduced 2-Cys Prxs might be able to directly reduce an oxidized target protein with their peroxidatic Cys (Fig. 6A, step 1) by forming a transient mixed disulfide with the TP. In the next step, the resolving Cys of the Prx might open this disulfide bond, and the reduced TP would be released (step 2). Step 2 could also be achieved with Trx reducing the disulfide bond. We recently confirmed with SPR that Trx is indeed able to attack a disulfide bridge introduced at the C_P_ of 2-Cys Prx (unpublished results). In our present experiments, using resolving Cys mutants, the former transient mixed disulfide between C_P_ and the TP would be stable since the resolving Cys is missing. Targeted proteins are therefore ‘trapped’ and can be eluted by the reducing agent DTT.

Proteins caught with the peroxidatic Cys mutants of Prx in our experiments might reflect a second mechanism for target protein reduction *in vivo* (Fig. 6B). Here, the C_P_ of wt Prx might be hyperoxidized to sulfinic acid (step 1). The still reduced C_R_ might then build a mixed disulfide with the TP (step 2), which redoxins could subsequently reduce (not shown). This mechanism is rather likely since we caught more TPs with C_P_ mutants than with C_R_ mutants (see Figs 2 and 3 and Tables 1, 2, S1 and S2). In our eyes, this adds a new dimension to the mechanistic principles and the versatility of Prx-based redox relays.

In addition to the C_P_ hyperoxidation described above, a sulfenic acid formation at the C_R_ might occur (Fig. 6C, step 1). In this scenario, the C_R_ could form a mixed disulfide with a former reduced target protein. This complex could serve as an oxidizing signal altering the properties of the TP or even protecting it from further damage. When the cell has recovered from an oxidative challenge, the disulfide could be resolved by downstream (reduced) redoxins. We recently confirmed via SPR that Trx is indeed able to attack a disulfide bridge introduced at the C_R_ of 2-Cys Prxs (unpublished results).

A second scenario leading to TP oxidation might involve both active site cysteines present as sulfenic acids. The oxidized C_P_ might then be reduced by a TP (not shown). However, since the simultaneous formation of sulfenic acids at both active site cysteines in 2-Cys Prxs has not yet been described, and since we caught only a few proteins with the *Pf*Prx1a resolving Cys mutant, this mechanism is less likely. Finally, proteins other than thioredoxin could reduce *in vivo* the reduction of the disulfide between C_P_ and C_R_ that occurs during the catalytic cycle of 2-Cys Prx wt. This would also lead to oxidation of the TP. The respective intermediates would not be caught with the Prx wt in our experiments; however, they might partially resemble TP interacting with C_P_ or C_R_ mutants.

Notably, many proteins interacting with our cytosolic or mitochondrial *Pf*Prx were not located in the same subcellular compartment and are therefore listed separately in the figures and tables of this manuscript. These interaction partners are nonetheless of great interest since they might represent target proteins of Prxs in other compartments^57^.

### Target proteins of Prx and present knowledge on their redox regulation

Several proteins captured with *Pf*Prx1a in our study have indeed been previously reported to be prone to redox modulation. However, a redox interaction or regulation by Prxs has hardly been described. For example, 40S ribosomal protein S23 and 40S ribosomal protein S21 (RPS21) from *Schizosaccharomyces pombe* were found to be oxidized in a thiol-labeling approach to characterize the disulfide proteome of fission yeast^58^. Lysine-tRNA ligase (KRS1) captured in our study was characterized to be peroxide sensitive in *Saccharomyces cerevisiae*^59^. Moreover, Trx1 was shown to redox-regulate heat shock protein 40 from mice (DnaJb5). DnaJb5, reduced by Trx1, is able to form a complex with class II histone deacetylases 4 (HDAC4) via intramolecular disulfide bonds in order to hinder HDAC4 nuclear export and thereby inhibit cardiac hypertrophy after ROS-generated hypertrophic stimuli^60^. In investigations of Parkinson’s disease patients, cysteine oxidation was identified in ubiquitin carboxyl-terminal hydrolase, a deubiquitinating enzyme^61^, and cysteine modifications such as disulfide formation, *S*-nitrosylation, and *S*-glutathionylation can modify the ubiquitin proteasome system itself^62,63^. Protein phosphatase 2C (PP2C) from *Arabidopsis thaliana* (ABI1) was significantly inactivated when challenged with hydrogen peroxide via specific cysteine oxidation^64^. The translation initiation factor 4E (eIF4E) was reported to form intramolecular disulfide bridges in order to impede a proper translation as shown in wheat^65,66^. Protein disulfide isomerase (PDI) is able to maintain disulfides and reconstruct un/misfolded proteins^67,68^ and was interestingly also found as a Prx-interacting protein in our study. Along with other proteins linked to folding, the redox-dependent heat shock protein 70 (Hsp70) was captured. Hsp70 had already been identified to interact with Trx and Grx in plants^45,46,57,69–71^, depicting its redox linkage. Adriamycin, a ROS-releasing, potent anticancer drug in humans, was shown to inhibit enolase, the enzyme that catalyzes the conversion of 2-phospho-D-glycerate to phosphoenolpyruvate in the penultimate step of glycolysis^72^. Furthermore, the enolases of *Arabidopsis thaliana* and *Mesernbryanthemum tallinum* were found to be redox-regulated by the Trx system^73^. Since *P. falciparum* enolase was captured in our pull-down assay, regulation in malaria parasites is likewise conceivable. Finally, a redox regulation of *S*-adenosyl-L-homocysteine hydrolase depending on Grx^51^ and Trx^47^ has been reported. Combining our present data on Prx interaction with existing knowledge on redox regulation and disulfide-mediated interaction of our TPs with other proteins will allow further understanding of the flow of reducing and oxidizing signals through the cell. It will also serve as a basis for studying functionally relevant redox interactions in molecular detail.

Identified proteins interacting with *Pf*Prx1m are involved in lipid metabolism, protein folding, redox control, and energy metabolism (clustered and summarized in Table 2). All TPs identified contain at least one cysteine residue, allowing for an intermolecular disulfide with *Pf*Prx1m in the experiment. The respective metabolic functions of the captured proteins are summarized in Table 2. Other authors have already described the redox modulation of some of these proteins in humans. Human diacylglycerol kinase (DGK), e.g., reduces the activity of the diacylglycerol (DAG) protein kinase C (PKC), an enzyme involved in the pathophysiology of diabetic nephropathy. It could be shown that antioxidants such as D-α-tocopherol and probucol can restore the strong glucose-induced decrease in DGK activity and that H_2_O_2_ mediates a downregulation of DGK activity, strongly suggesting a redox-dependent regulation of DGK^74^. Since the pull-down assay implemented here revealed DGK as a potential interacting partner for *Pf*Prx1m, the Prx might have a function in maintaining DGK activity. The electron transport chain in combination with F_1_F_O_-ATP synthase produces cellular ATP^75^. F_1_F_O_-ATP synthase shows a high number of oxidative post-translational modifications (Ox-PTMs) at cysteines of the α- and γ-subunits^76–79^, which were found to be negatively correlated to conformational changes and ATP synthase hydrolytic activity (summarized in^44^). Since the aforementioned proteins were captured with *Pf*Prx1m in triplicate in our study and have already been reported to be redox regulated in other organisms, *Pf*Prx1m might play a role in regulating this important step of energy metabolism in *Plasmodium falciparum*.

## Conclusions

In order to further validate the results of our pull-downs from cell extracts we tested the interaction between *Pf*Prx1a wt and recombinant *Pf*LDH and *Pf*SAHH. Both proteins were indeed confirmed to be disulfide-based binding partners of *Pf*Prx1a (see Fig. 4). Furthermore, the enzyme activities of *Pf*LDH and *Pf*AK were found to increase strongly (for AK significantly) in the presence of 25 µM *Pf*Prx1a (Fig. 5). In this context, it should be mentioned that *Pf*LDH, HSP70, disulfide isomerase, and SAHH were also captured in pull-down experiments with Trx, Grx, and Plrx^47,48^, suggesting an interplay or even synergy between Prxs, their respective redoxin, and the target protein. Based on our results and the previously reported redox sensitivity of various captured target proteins, the functional importance of Prx-based redox relays in eukaryotic cells can be assumed to be much higher than expected so far. The Prx-based redox regulation of the TPs identified in our study, the regulation of distinct metabolic pathways, and the crosstalk with other signaling pathways should by studied in further detail.

## Materials and Methods

### Materials

All reagents used were of the highest purity available. NADPH was from Biomol, Hamburg. Imidazole, IPTG, and 1,4-dithiothreitol (DTT) were obtained from Roth, Karlsruhe, and Ni-NTA agarose from Invitrogen, Karlsruhe. PCR primers were from MWG-Biotech, Ebersberg; the vector pQE30 and the *E. coli* host strain M15 were from Qiagen, Hilden. CNBr-activated Sepharose 4B (Amersham) was purchased from GE Healthcare, Munich.

### *Plasmodium falciparum* cell culture and preparation of the parasite lysate

The chloroquine-sensitive strain 3D7 was maintained in cell culture according to Trager and Jensen with modifications^80^. In brief, parasites were grown in red blood cells (A+) at 5% hematocrit with a parasitemia no greater than 8% at a steady 37 °C, purged with a gas mixture containing 3% O_2_, 3% CO_2_, and 94% N_2_. Parasite growth was monitored via Giemsa staining. To ensure synchronous parasites, sorbitol synchronization was performed according to Lambros and Vanderberg^81^. Once parasites became trophozoites, saponin lysis was conducted to remove red blood cells and obtain the parasite pellet^82^. The pellets were stored at −80 °C until use.

For parasite lysate preparation, a pellet from eight large culturing plates (45 mL each) of *P. falciparum* 3D7 with 5% hematocrit and 7% parasitemia was blended with 1.5 mL wash buffer (100 mM Tris, 500 mM NaCl, pH 8.0) in an Eppendorf cup, which was additionally sealed with Parafilm. The cup was frozen in liquid nitrogen for 2 min and thawed in lukewarm water until the suspension was liquid again. The freeze and thaw cycle was repeated four times in order to lyse the parasite cells. Subsequently, the samples were transferred into centrifuge tubes and spun down at 136,000 g for 30 min at 4 °C in an ultracentrifuge (Beckmann). The protein concentration of the resulting parasite lysate was determined with the Bradford assay at 595 nm^83^. The protein concentration of the parasite lysate was between 8–10 mg/mL.

### Cloning and site-directed mutagenesis of peroxiredoxins

*Pf*Prx1a (PlasmoDB ID: PF3D7_1438900, thioredoxin peroxidase (1), and *Pf*Prx1m (PlasmoDB ID: PF3D7_1215000, thioredoxin peroxidase (2) were cloned as described previously^32,84^. The production of the *Pf*Prx1a C170S mutant was also previously described^85^. The *Pf*Prx1a C_P_ mutant was generated via site-directed mutagenesis using *Pf*Prx1a wild type (pQE30/*Pf*Prx1a) as a template, Pfu polymerase (Promega Corp.), and these mutagenesis primers: *Pf*Prx1a^C50S^, 5′-TTTTACGTTTGTATCTCCATCTGAAATC-3′. For mutations of active site cysteines of *Pf*Prx1m, the following primers were used: *Pf*Prx1m^C67S^, 5′-CTATACCTTTGTCTCTCCAACCGAAAT-3′; *Pf*Prx1m^C187S^, 5′-TCTGGTGAA GTTTCTCCGATCAATTG-3′. Methylated, non-mutated template plasmids were digested with DpnI, and the correct mutation was confirmed via sequencing. For control experiments, recombinant *Pf*SAHH and *Pf*LDH were produced as previously described^65,86^.

### Heterologous overexpression and purification of peroxiredoxins

For pull-down experiments, the 2-Cys peroxiredoxins (*Pf*Prx1a and *Pf*Prx1m), their resolving cysteine mutants (*Pf*Prx1a^C170S^ and *Pf*Prx1m^C187S^), and their peroxidatic cysteine mutants (*Pf*Prx1a^C50S^ and *Pf*Prx1m^C67S^) were used. Additionally, 2-Cys *Pf*Prxs lacking their peroxidatic and resolving cysteines (*Pf*Prx1a^C50S/C170S^ and *Pf*Prx1m^C67S/C187S^) were employed as bait for potential mixed disulfides via non-active site cysteine residues with parasite proteins.

Recombinant *Pf*Prx1a wild type, *Pf*Prx1a^C50S^, *Pf*Prx1a^C170S^, and *Pf*Prx1a^C50S/C170S^ were heterologously overexpressed with an N-terminal His_6_-tag in competent *E. coli* M15 cells using the pQE30 vector. Cells were grown in lysogeny broth (LB) medium containing 50 µg/mL kanamycin and 100 µg/mL carbenicillin at 37 °C while shaking up to an OD_600_ of 0.6. Gene expression was induced with 1 mM isopropyl-D-thiogalactopyranoside (IPTG) for 4 h at 37 °C. His-tagged *Pf*Prx1m wild type, *Pf*Prx1m^C67S^, *Pf*Prx1m^C187S^, and *Pf*Prx1m^C67S/C187S^—all in pQE30—were transformed into competent *E. coli* M15 cells. Cells were grown in terrific broth (TB) medium containing 50 µg/mL kanamycin and 100 µg/mL carbenicillin at 37 °C while shaking up to an OD_600_ of 0.6. Gene expression was induced by 0.5 mM IPTG for 24 h at RT.

All cells were harvested via centrifugation (4 °C, 12,000 g, 15 min) and re-suspended in 50 mM Na_2_HPO_4_, 300 mM NaCl buffer pH 8.0, containing pepstatin (150 nM), cystatin (4 nM), and PMSF (100 nM) and stored at −20 °C. For cell disruption, the suspension was thawed, 1 mg/ml lysozyme and DNase I were added, and cells were lysed for 1 h on ice, followed by sonication at 4 °C for 3 cycles at 70% of maximum power followed by centrifugation (25,000 g, 4 °C, 30 min). Proteins in the supernatant were purified via a Ni-NTA (nickel-nitrilotriacetate) affinity column in the presence of 0.5 mM 1,4-dithiothreitol (DTT) to maintain a reduced state in the respective proteins. Proteins were eluted by an imidazole gradient, and the eluted fractions were monitored on Coomassie Brilliant Blue-stained SDS gels. Next, the proteins were brought to a final concentration of about 5 mg/ml by using Vivaspin columns (Sartorius, Göttingen) and were stored at 4 °C.

Protein concentration was determined with the Bradford assay at 595 nm and spectrophotometrically at 280 nm using the calculated extinction coefficients of the proteins (*Pf*Prx1a: Ɛ_280_ = 21,700 M^−1^ cm^−1^; *Pf*Prx1m: Ɛ_280_ = 21,800 M^−1^ cm^−1^). The amount of protein obtained from one liter of *E. coli* cell culture averaged between 1–10 mg, depending on the respective protein and batch.

In preparation for the pull-down experiments, we showed that the 6xHis-tagged *Pf*Prx1a and *Pf*Prx1m are in a decameric state when reduced with DTT and are functionally active. This is true for both proteins in purification buffer as well as in the coupling buffer used for the pull-down experiments. In spite of this, it cannot be fully excluded that the 6xHis-tag could potentially influence the interaction with target proteins.

### Pull-down implementation

To identify interacting partners that are addressed for H_2_O_2_ signal transduction and/or reduction by plasmodial peroxiredoxins *in vitro*, a pull-down assay based on disulfide-bonded reaction intermediates was developed. For this purpose, the respective Prx was immobilized on CNBr-activated Sepharose 4B beads via amine coupling and was then incubated with parasite lysate from the *Plasmodium falciparum* 3D7 strain containing soluble potentially interacting proteins (Fig. S1). All experiments were conducted in three independent biological replicates. Eluted proteins were visualized on silver-stained SDS gels (Fig. S1) and analyzed via mass spectrometry.

CNBr-activated Sepharose 4B beads (Amersham) that had been lyophilized in the presence of additives were purchased; the additives were washed away with a low pH buffer (pH 3) before protein coupling. The low pH also preserves the activity of the reactive CNBr groups (cyanate esters). These groups are able to react with primary amines to couple proteins onto the agarose matrix, building isourea derivatives immobilized at the Sepharose beads. Unsaturated groups were blocked with buffer containing the small primary amine Tris. To remove ligands that had been bound ionically to the immobilized protein, the matrix was washed with low and high pH buffers containing 500 mM NaCl.

### Ligand coupling

5.7 mg of lyophilized CNBr-activated Sepharose 4B beads were suspended in 20 µL of 1 mM HCl. The swollen material was washed for 10 min with 800 µL 1 mM HCl followed by centrifugation for 90 sec at 12,045 g in a MiniSpin centrifuge. The supernatant was discarded, and the beads were washed again for 5 min with 400 µL of 1 mM HCl. After centrifugation for 90 sec at 12,045 g, the supernatant was discarded again. For ligand coupling, the respective protein was dialyzed three times for 30 min in coupling buffer (100 mM NaHCO_3_, 500 mM NaCl, 0.5 mM DTT, pH 8.3). 2 mg of reduced protein in coupling buffer was blended with washed beads and slightly swiveled for 1 h at 4 °C using a hybridization oven (OV 2, Biometra). The mixture was centrifuged again for 90 sec at 12,045 g, and the supernatant was discarded. The excess ligand that had not bound to the Sepharose beads was washed away with three cycles of inverting the material with 200 µL of coupling buffer and 90 sec of centrifugation at 12,045 g. Unsaturated groups were blocked with 200 µL of blocking buffer (100 mM Tris-HCl, 0.5 mM DTT, pH 8.0) for 2 h at RT. After blocking, the medium was washed with three cycles of 200 µL pH shift buffer I (100 mM C_2_H_3_NaO_2_, 500 mM NaCl, 0.5 mM DTT, pH 4.0), alternating with 200 µL pH shift buffer II (100 mM Tris-HCl, 500 mM NaCl, 0.5 mM DTT, pH 8.0). After each step of the pH seesaw, the mixture was centrifuged for 90 sec at 12,045 g, and the supernatant was discarded. To keep the decameric state of the recombinant peroxiredoxins, all immobilization steps were performed in the presence of 0.5 mM DTT. Directly before the cell lysates were added, the beads were washed with buffer II without DTT.

### Pull-down assay

Approximately 6 mg of parasite lysate proteins were incubated with the loaded beads (20 µL) at RT for 2 h while slightly swiveling the sample on a rocker. The beads were repeatedly washed with 200 µL wash buffer (100 mM Tris, 500 mM NaCl, pH 8.0) in order to remove unbound parasite proteins. Washing was repeated until protein could no longer be observed in the supernatant by determining the protein concentration at 280 nm in a UV cuvette using a biophotometer (Eppendorf). To elute covalently bound parasite proteins, the beads were incubated with 70 µL of wash buffer containing 10 mM of DTT for 30 min at RT while occasionally inverting the mixture. After incubation, the cup was centrifuged again, and the supernatant was stored at −20 °C until used for MS analysis.

To visualize the outcome of the pull-down assay, SDS-PAGE was conducted with aliquots of parasite lysate, different washing steps, and the eluate (Fig. S1). One µL of parasite lysate was mixed with 15 µL of 1 × sample buffer; 5 µL of the first washing step was mixed with 15 µL of 1 × sample buffer; 15 µL of an intermediate and 15 µL of the last washing step were mixed with 4 × sample buffer; and 15 µL of the eluate was mixed with 4 × sample buffer. Since the concentration of proteins in the eluate fraction was expectedly very low, silver staining was carried out according to the manufacturer’s protocol (Pierce Silver Stain Kit, Thermo Scientific).

### Sample preparation for mass spectrometry analysis

For MS analysis, the eluates were sent in a dry ice parcel to The Scripps Research Institute in La Jolla, California. There, samples were mixed with 60 µL of 200 mM Tris and 57 mg of solid urea. 30 µL of Ni-NTA agarose, which had been washed and equilibrated in 100 mM Tris, pH 8.5, was added to each sample to remove the recombinant His-tagged *Pf*Prxs. The slurry was shaken at RT for 1 hour. Samples were centrifuged at top speed for 30 minutes, and the supernatant was transferred to a new microcentrifuge tube. Supernatants were spun again for 20 minutes, removed, and then moved to a new tube. Samples were reduced with 6 µL 100 mM TCEP and alkylated with 6 µL of 250 mM IAA. 1 µg of endo-LysC was added, and the reaction was allowed to proceed for 4 hours at 37 °C. Samples were diluted with 360 µL 100 mM Tris pH 8.5, and 2 µg of trypsin was added. Samples were shaken at 37 °C for 18 hours, quenched with 25 µL of formic acid, and spun for 20 minutes.

### Mass spectrometry analysis

After transfer to another tube, the supernatant was filtered through a 100 µm capillary column with a frit. Filtered supernatants were pressure-loaded onto a fused silica microcapillary column containing 2.5 cm of Partisphere strong cation exchanger (Whatman) followed by 2.5 cm of 10 cm Aqua C18 (Phenomenex) packed into a 250 µm inside diameter (i.d.) capillary (Polymicro Technologies) with a 1 cm frit. The column was washed for 60 min with buffer A. After washing, a 100 µm i.d. capillary with a 5 µm pulled tip packed with 15 cm of 3 µm Aqua C18 material (Phenomenex) was attached via a union, and the entire split column was placed in line with an Agilent 1100 quaternary high-performance liquid chromatography (HPLC) and analyzed using a nine-step separation. The buffer solutions used for separation were 5% acetonitrile/0.1% formic acid (Buffer A), 80% acetonitrile/0.1% formic acid (Buffer B), and 500 mM ammonium acetate/5% acetonitrile/0.1% formic acid (Buffer C). Step 1 consisted of a 90 min gradient from 0% to 100% buffer B. Steps 2–9 had the following profile: 10 min of X% buffer C, a 15 min gradient from 0% to 5% buffer B, and a 95 min gradient from 15% to 100% buffer B. The 10 min buffer C percentages (X) were 10%, 20%, 30%, 40%, 50%, 60%, 70%, and 100%, respectively, for the nine-step analysis. As peptides were eluted from the microcapillary column, they were electrosprayed directly into an Orbitrap Velos mass spectrometer (ThermoFisher) with the application of a distal 2.4 kV spray voltage. A cycle of one full-scan mass spectrum (400–1,800 m/z) followed by 15 data-dependent tandem mass spectrometry spectra at 35% normalized collision energy was repeated continuously throughout each step of the multidimensional separation. The application of mass spectrometer scan functions and HPLC solvent gradients was controlled via the XCalibur data system.

### Mass spectrometry data analysis

Tandem mass spectra were analyzed with an Integrated Proteomics Pipeline (IP2; Integrated Proteomics Applications, Inc., San Diego, CA. http://www.integratedproteomics.com) using ProLuCID^87^ and DTASelect2.0^88,89^. Spectrum raw files were extracted into MS1 and MS2 files using RawExtract 1.9.9 (http://fields.scripps.edu/downloads.php)^90^, and tandem mass spectra were searched against the PlasmoDB database (release date 03/30/15). To accurately estimate peptide probabilities and false discovery rates, a decoy database containing the reversed sequences of all proteins appended to the target database^91^ was used. Tandem mass spectra were matched to sequences using the ProLuCID algorithm with 50 ppm peptide mass tolerance^92^, where at least 2 peptides per protein had to pass the filtering process with a minimum peptide length of 6 aa. The search space included all peptide candidates without restriction to tryptic cleavage that fell into the mass tolerance window. Modifications of +57.02146 on C owing to IAA treatment as part of the sample preparation were considered to be a potential modification. The validity of peptide/spectrum matches was assessed according to Wang *et al*.^92^ with a protein confidence of 97% as the minimum threshold and a peptide delta mass limitation of 15 ppm maximum, resulting in a protein false discovery rate below 1.0%.

To account for unspecific protein binding of parasite lysate to the CNBr-activated Sepharose 4B beads, the immobilization of bait proteins to the beads was mimicked with water during the pull-down procedure, which was also conducted in triplicate. Parasitic proteins captured with empty beads were subtracted from all ensuing results. Tables S1 and S2 provide an overview of proteins captured with the wild type enzymes, the peroxidatic or resolving cysteine mutants of *Pf*Prx1a and *Pf*Prx1m, and their double active site mutants. In the supplementary datasets of *Pf*Prx1a and *Pf*Prx1m, all raw files are provided.

In our pull-downs, MS identified a few human proteins in the elution fraction, which were treated as contaminants and were excluded from further data analysis.

### Pull-down validation experiments

To confirm covalent binding of selected TPs identified in cell extracts with recombinant proteins, *Pf*LDH^86^ and *Pf*SAHH^47^ were chosen as representative interaction partners of *Pf*Prx1a wt. For these experiments, 2 mg of reduced recombinant *Pf*Prx1a wt was coupled to CNBr-activated Sepharose 4B beads (Amersham) as described above in the pull-down implementation chapter and was kept in a reduced state with 0.5 mM DTT during all immobilization steps (see above). After a final washing of the beads in the absence of DTT, 8 mg purified recombinant *Pf*LDH wt or *Pf*SAHH wt (not pre-reduced) were incubated with the loaded beads. As described above for the pull-downs with cell lysates (please see section above for details), the beads were extensively washed, and bound protein was eluted with 10 mM of DTT and visualized via silver staining on SDS gels.

### Functional implications of interaction with Prx

To study the influence of *Pf*Prx1a on the enzyme activities of selected target proteins, spectrophotometric assays were carried out (two independent experiments). For this, GST^93,94^, LDH, and AK^95^ were produced as previously described. We then freshly purified *Pf*Prx1a, reduced it with 1 mM TCEP, desalted the samples via Zeba spin desalting columns, and split them into two fractions. One fraction was immediately oxidized with 1 mM H_2_O_2_ and again desalted. Then, 25 µM of reduced or oxidized Prx was incubated for 5 min with the non-prereduced enzymes GST, LDH, or AK, and the respective functional assays were conducted (see below). Control samples contained (instead of Prx) buffer only or 25 µM of BSA (to account for simply stabilizing effects).

Assays were carried out at 25 °C using a Hitachi U-2001 spectrophotometer (total assay volume 500 µL). For enzyme activity calculations, the extinction coefficients at 340 nm of CDNB conjugated glutathione (9.6 mM^−1^ cm^−1^) or NADH (6.22 mM^−1^ cm^−1^) were used. *Pf*GST activity (final concentration 1.57 µM) was measured in 100 mM HEPES, 1 mM EDTA, pH 6.5, and 1 mM reduced glutathione (GSH). The reaction was started by adding 0.5 mM 1-chloro-2,4-dinitrobenzene (CDNB), and the initial ΔA/min was monitored at 340 nm^93^. *Pf*LDH activity (final concentration 16 nM) was measured in 100 mM sodium phosphate buffer, pH 7.5, and 100 µM NADH. The reaction was started by adding 5 mM pyruvate, and the initial ΔA/min was monitored at 340 nm^86^. The activity of *Pf*AK (final concentration 33 nM) was determined in 110 mM TEA, 1.5 mM MgCl_2_, and 60 mM KCl, pH 7.2. Substrates and enzymes for the coupled assay were used as follows: 200 µM NADH, 10 U/ml LDH (from rabbit muscle, Roche), 400 µM phosphoenolpyruvate (PEP), 10 U/ml pyruvate kinase (from rabbit muscle, Roche), 1 mM ATP and 2 mM AMP^96^. The reaction was started with AMP, and the initial ΔA/min was monitored at 340 nm.

## Supplementary information

Supplementary Figure 1

Supplementary Table 1

Supplementary Table 2

Supplementary Dataset PfPrx1a

Supplementary Dataset PfPrx1m

## Data Availability

The authors declare that the data supporting the findings of this study is available within the paper and its supplementary information files.
