# Supplementary material for: The interactome of 2-Cys peroxiredoxins in Plasmodium falciparum
Source: Sci Rep. 2019 Sep 19;9:13542. doi: 10.1038/s41598-019-49841-3 (PMC6753162; doi:10.1038/s41598-019-49841-3)

The interactome of 2-Cys peroxiredoxins in *Plasmodium falciparum*

Christina Brandstaedter[1], Claire Delahunty[2], Susanne Schipper[1], Stefan Rahlfs[1], John R. Yates III[2], Katja Becker[1]*

[1] Biochemistry and Molecular Biology, Interdisciplinary Research Centre, Justus Liebig University Giessen, Heinrich Buff Ring 26-32, 35392 Giessen, Germany

[2] Chemical Physiology, The Scripps Research Institute, 10550 North Torrey Pines Rd., SR11, La Jolla, CA 92037, USA

*Corresponding author: E-mail: katja.becker@uni-giessen.de

KEYWORDS: Interactome – Malaria parasite – Peroxiredoxins – *Plasmodium falciparum* – Redox signaling – Sensor – Transducer

Supplementary Figure 1. Elution profile of 2-Cys *Pf*Prxs during pull-down assays. Representative elution profiles of the pull-down experiment were separated on a 12% SDS-PAGE and visualized with silver staining after gel electrophoresis. L: protein ladder, Ly: parasite lysate, W: number of washing steps, E: eluate with 10 mM DTT.

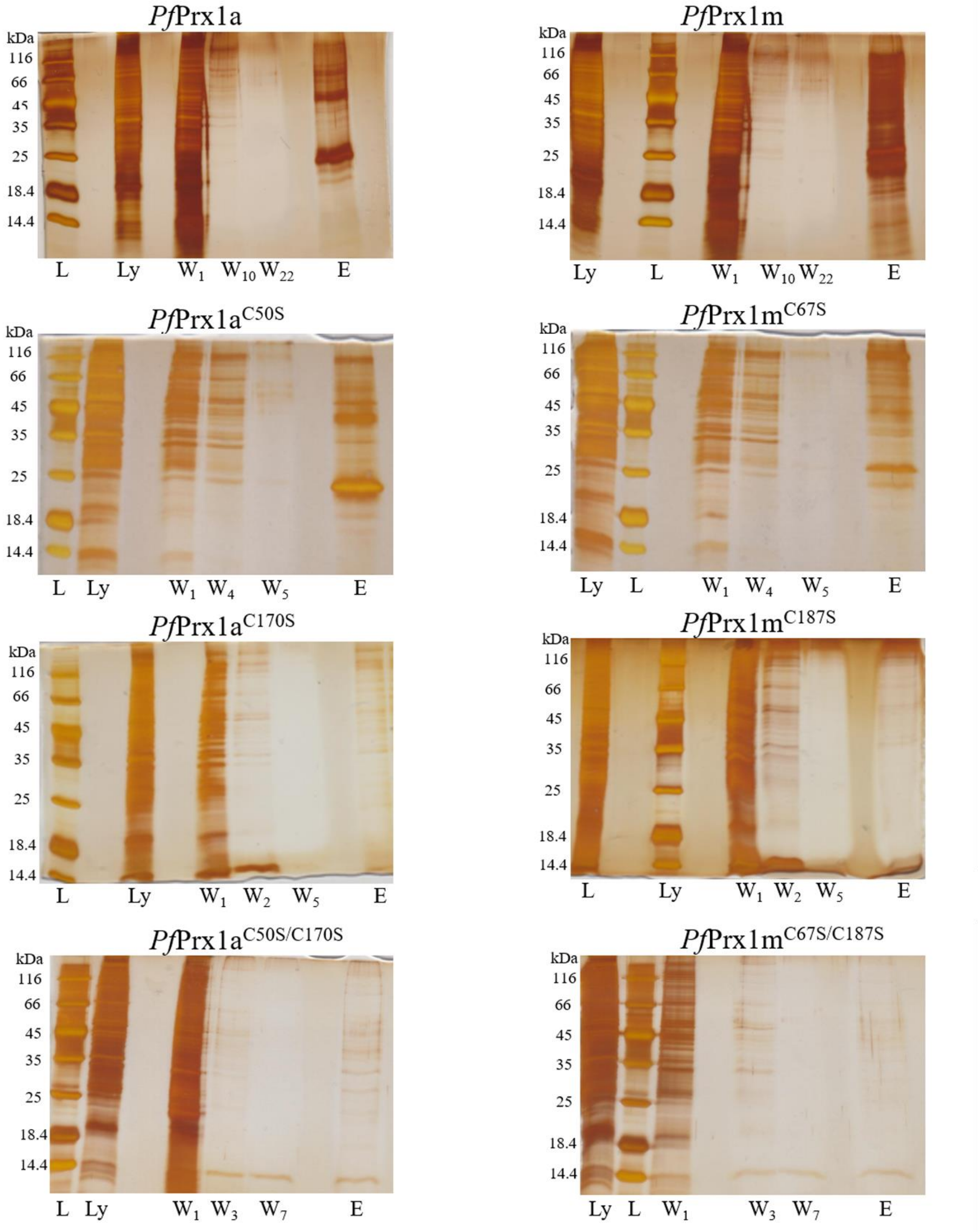

Supplement: Supplementary file 1 — Supplementary Figure 1 [file 41598_2019_49841_MOESM1_ESM.pdf]
